# Live birth after transfer of a tripronuclear embryo: An intracytoplasmic sperm injection as a combination of microarray and time-lapse technology

**DOI:** 10.4274/tjod.45144

**Published:** 2016-06-15

**Authors:** Ender Yalçınkaya, Alev Özay, Elif G. Ergin, Zeynep Öztel, Hakan Özörnek

**Affiliations:** 1 Eurofertil In Vitro Fertilization Center, Embryology Laboratory, İstanbul, Turkey

**Keywords:** Tripronuclear, Microarray, euploid

## Abstract

Although around 1-4% of human zygotes have been found to be tripronuclear, there is little information about the subsequent development and chromosomal composition of embryos that derive from these zygotes. Herein, we report a pregnancy and subsequent delivery of a healthy newborn after the transfer of a blastocyst that developed from a tripronuclear zygote that had a euploid microarray result.

## INTRODUCTION

During assisted reproduction treatments, successful fertilization is determined through the presence of two visible pronuclei in the cytoplasm of the oocyte on the first day. Also, two common abnormal fertilization patterns have been reported in clinical practice. These are 1 pronucleus (PN) and 3PN zygotes. It is known that triploid human embryos are associated with spontaneous abortions after implantation^(1)^. For this reason, it is believed that it is crucial to recognize 3PN formation in the early period and not to transfer the embryos that develop from 3PN oocytes during in vitro fertilization (IVF) treatment.

Aneuploidies are commonly observed in early human embryos^(2,3)^. Preimplantation genetic diagnosis (PGD) has been applied as a method in assisted reproductive technology to select genetically normal embryos for transfer that have the highest implantation potential. It is generally recommended for the people who are referred to IVF clinics with an etiology such as repeated implantation failure, recurrent pregnancy loss, and advanced age. After the introduction of microarrays in assisted reproduction practice, physicians and embryologists began to obtain more detailed information about the whole genomic constitution of the embryos.

In this case report, we aimed to present a healthy delivery by a woman whose transferred embryo was developed from a 3PN zygote until the blastocyst stage and yielded a euploid result with microarray analysis.

## CASE REPORT

**Figure 1:**
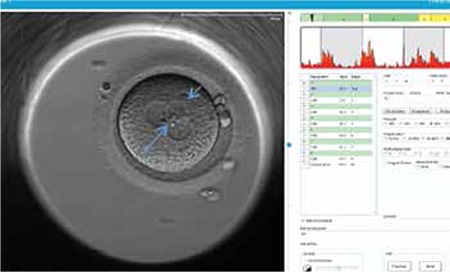
The image of 3PN embryo on embryoscope screen at day 1. Blue arrows indicate the exact location of third pronucleus

**Figure 2:**
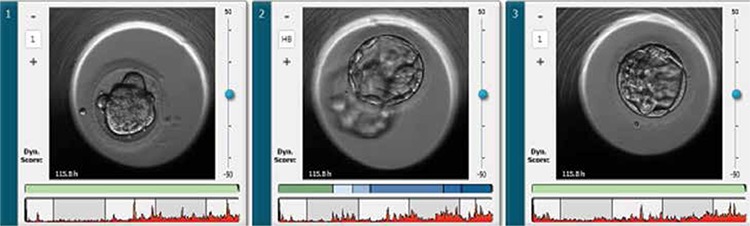
All three embryos at day 5, 1) 2PN (45,XX, -16) 2) 3PN (46,XX) (euploid) 3) 47,XY, +6

A woman aged 36 years was admitted to our IVF clinic with the etiology of diminished ovarian reserve. She had experienced two previous pregnancies; however, both pregnancies were terminated with missed abortions at around 13 weeks due to trisomy XXY. All sperm parameters were within normal range based on World Health Organization (WHO) 2010 criteria. The patient was offered genetic testing for a possible aneuploidy along with intrcytoplasmic sperm injection (ICSI) treatment due to the previous abortions, which she accepted. She underwent an antagonist protocol with the administration of 450 IU recombinant follicle stimulating hormone (Gonal F, Merck Serono, Italy) for 7 days. The antagonist (Cetrotide, 0.25 mg) was administered after the observation of a leading follicle of 15 mm. Human choriogonadotrophin (hCG) was administered when more than three ≥17 mm oocytes were seen. Three oocytes were collected 36 hours after hCG (Ovitrelle, Merck Serono, Italy) administration. All oocytes were at metaphase II (MII) stage and they all underwent ICSI. ICSI is the preferred method of fertilization in our clinic irrespective of semen parameters due to its higher fertilization rates. A fertilization check was performed 17 hours after microinjection. Two oocytes were normally fertilized (2PN) and one was abnormally fertilized (3PN). All zygotes were cultured in ISM1 medium (Medicult, Origio, Denmark) until the end of day 2 in Blast Assist medium (Medicult, Origio, Denmark) until the blastocyst stage in a closed incubation system including time-lapse photography (Embryoscope, Unisense Fertilitech, Denmark) under the culture conditions of 37 °C, 6.0% CO_2_ and 7.0% O_2_. We use lower oxygen concentrations for embryo culture in our laboratory becayse there are higher blastocyst development and clinical success rates reported in the literatüre^(4)^. All three embryos were good quality at day 3 (including ≥6 even blastomeres and no fragmentation). One single blastomere was biopsied from all day 3 embryos and sent for genetic analysis. All 24 chromosomes were screened using a comparative genomic hybridization array following genome amplification in single cell. The genetic results arrived in the morning of day 5, and that only embryo that developed was from 3PN zygote and it was found to be normal (euploid) based on the report. The patient was informed about all possible consequences of transferring an embryo developed from a 3PN zygote and she gave consent for its transfer. The embryo cleaved properly based on the expected timing intervals of Meseguer’s hierarchical model, and reached hatching blastocyst stage on day 5^(5)^. An image of the 3PN embryo that was taken from the embryoscope screen is shown in Figure 1. One of the other embryos (2PNs) also developed to blastocyst stage and the other arrested at the compaction stage (Figure 2). The microarray results of the embryos are given in Table 1. A hatching blastocyst was transferred to the patient by the common decision of the geneticist, embryologist, physician, and the patient. The patient was considered as pregnant after obtaining a βhCG result of 652 mIU/mL after 10 days following embryo transfer and the quantitative hCG result doubled after two days. The patient delivered a healthy baby girl after 39 weeks by cesarian section. The weight of the baby was 3410 gr and her height was 51 cm at birth.

## DISCUSSION

Although around 1-4% of human zygotes were found to be tripronuclear, there is little information about subsequent development and chromosomal composition of embryos that derive from these zygotes^(6)^. The reason is that these tripronuclear zygotes are generally discarded in routine IVF practice because they are believed to be genetically abnormal. Despite this general belief, it was shown in some studies that some of these tripronuclear human oocytes do not always develop into triploid embryos. Kola et al.^(6)^ showed that a large majority of tripronuclear human oocytes do not develop into triploid embryos, and that the first cleavage is the critical stage that affects the subsequent chromosomal constitution of tripronuclear human oocytes. In some other studies that evaluated the chromosomal composition of 3PN zygotes and embryos developed from these zygotes, various amounts of them were found to be diploid^(7)^. These data are one of the reasons why we performed blastomere biopsy also to the 3PN embryo in this case. The second reason was the few embryos with which we had to work.

3PN formation has been acounted for by two main features: Dispermic fertilization and nonextrusion of the second polar body^(3)^. After the introduction of ICSI to the assisted reproduction armoury, it has become the preferred method of many IVF clinics because of its high fertilization and comparable pregnancy rates for male factor infertility problems and other etiologies. We could rule out the possibility of dispermic fertilization in this case because it is the only technique routinely used in our IVF clinic. Therefore, nonextrusion of the second polar body was the only possible explanation for this 3PN formation.

In the review by Munne and Cohen^(3)^ on chromosome abnormalities in human embryos, the different percentages of diploid 3PN embryos observed in different centers were attributed to differences in recording vacuoles and pronuclei. In our study, our embryo was cultured in a time-lapse incubator (Embryoscope, Unisense Fertilitech, Aarhus, Denmark) and we had the opportunity to check the pronuclear status of the embryo repeatedly by four experienced embryologists; it was determined that the third formation included pronucleolar bodies inside. We can also suggest that it would be better to put oocytes into a time-lapse system directly after ICSI such that the details of 3PN formations can be seen. The reason for this suggestion is based on our experiences with time lapse, which shows that 2PN zygotes may sometimes later appear as 3PN following fragmentation of one of its pronuclei.

In previous studies, 25-62% of human day 3 preimplantation embryos were found chromosomally mosaic and the average percentage of aneuploid blastomeres in the mosaic embryo ranges between 40-52%^(8)^. The genetic results obtained from a single blastomere may not be an exact representation of the whole embryo because of the high ratio of mosaicism in preimplantation day 3 embryos. Thus, two approaches may improve PGD accuracy. The first is the biopsy of two blastomeres on day 3, and the second is biopsy at a more advanced development stage such as blastocyst. In an unselected population of human blastocysts, it could be expected that nearly 40% could be chromosomally abnormal^(9)^. Thus, it can be concluded that the development of an embryo up to the blastocyst stage does not mean that the embryo is genetically normal. Accordingly, the main limitation in our study seems to be the timing of biopsy. As such, we could not determine the final chromosomal constitution of the two blastocysts we obtained during the treatment. The reason why we performed day 3 biopsy was that the patient was living abroad and did not want to come back for a thaw cycle. In addition, the reason for performing only a single blastomere biopsy instead of two was the double financial burden of the microarray, which was not approved by the patient.

## CONCLUSION

As far we know, this is the first study to show time-lapse imaging of a 3PN embryo that was found to be euploid using microarray-based genetic testing, which reulted with a healthy newborn. We conclude that it would be useful to perform aneuploidy testing also for abnormally-fertilized oocytes during IVF treatment, especially for patients with a low number of embryos. However, further studies on this subject should focus on a day-5 biopsy to minimize the confounding effect of mosaicism.

## Figures and Tables

**Table 1:**
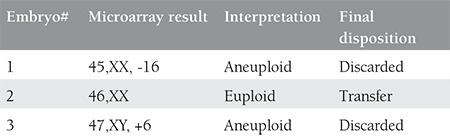
Microarray outcome of the biopsied blastomeres

## References

[1] Jacobs PA, Angell RR, Buchanan IM, Hassold TJ, Matsuyama AM, Manuel B (1978). The origins of human triploids. Ann Hum Genet.

[2] Harper JC, Coonen E, Handyside AH, Winston RM, Hopman AH, Delhanty JD (1995). Mosaicism of autosomes and sex chromosomes in morphologically normal, monospermic preimplantation human embryos. Prenat Diagn.

[3] Munne S, Cohen J (1998). Chromosome abnormalities in human embryos. Hum Reprod Update.

[4] Bontekoe S, Mantikou E, Wely M, Seshadri S, Repping S, Mastenbroek S (2012;11). Low oxygen concentrations for embryo culture in assisted reproductive technologies. Cochrane Database Syst Rev.

[5] Cruz M, Garrido N, Herrero J, Perez-Cano I, Munoz M, Meseguer M (2012). Timing of cell division in human cleavage-stage embryos is linked with blastocyst formation and quality. Reprod Biomed Online.

[6] Kola I, Trounson A, Dawson G, Rogers P (1987). Tripronuclear human oocytes: altered cleavage patterns and subsequent karyotypic analysis of embryos. Biol Reprod.

[7] Chen ZC, Yan J, Feng HL (2005). Aneuploid analysis of tripronuclear zygotes derived from in vitro fertilization and intracytoplasmic sperm injection in humans. Fertil Steril.

[8] Gonzalez-Merino E, Emiliani S, Vassart G, Vannin AS, Abramowicz M, et al (2003). Incidence of chromosomal mosaicism in human embryos at different developmental stages analyzed by fluorescence in situ hybridization. Genet Test.

[9] Magli MC, Jones GM, Gras L, Gianaroli L, Korman I, Trounson AO (2000). Chromosome mosaicism in day 3 aneuploid embryos that develop to morphologically normal blastocysts in vitro. Human Reprod.

